# A critical review of the evolution and interrelation of traumatic stress disorders

**DOI:** 10.1371/journal.pmen.0000385

**Published:** 2025-07-22

**Authors:** Ayah Hamadeh, Joseph El-Khoury, Julio Torales, Mia Atoui, Neil Aggarwal, Mike Campbell, Myrna Lashley, Joana Corrêa de Magalhães Narvaez, Audrey McMahon, Antonio Ventriglio, Rowalt Alibudbud, Padmavati Ramachandran, Helena Moura, Tarek Okasha, Rachel Tribe, Geraint Day, Afzal Javed, Albert Persaud, Dinesh Bhugra

**Affiliations:** 1 Department of Psychiatry, American University of Beirut Medical Center, Beirut, Lebanon; 2 Department of Psychiatry, United Arab Emirates University, Al Ain, Abu Dhabi, United Arab Emirates; 3 Facultad de Ciencias Médicas, Universidad Nacional de Asunción, San Lorenzo, Paraguay; 4 Universidad de Los Lagos, Osorno, Chile; 5 Embrace, Beirut, Lebanon; 6 Department of Psychiatry, Columbia University, New York City, New York, United States of America; 7 New York State Psychiatric Institute, New York City, New York, United States of America; 8 Faculty of Medical Sciences, University of the West Indies, Cave Hill, Barbados; 9 Division of TransCultural Psychiatry, Department of Medicine, McGill University, Montreal, Canada; 10 Universidade Federal de Ciências da Saúde de Porto Alegre, Porto Alegre, Brazil; 11 Doctors Without Borders, Amman, Jordan; 12 Department of Clinical and Experimental Medicine, University of Foggia, Foggia, Italy; 13 Department of Sociology and Behavioral Sciences, De La Salle University, Manila City, Philippines; 14 Department of Psychiatry and Behavioral Medicine, University of the Philippines - Philippine General Hospital, Manila City, Philippines; 15 Schizophrenia Research Foundation, Chennai, Tamil Nadu, India; 16 Faculty of Medicine, University of Brasilia, Brasilia, Brazil; 17 Department of Neuropsychiatry, Faculty of Medicine, Ain Shams University, Cairo, Egypt; 18 Department of Psychology & Human Development, University of East London, London, United Kingdom; 19 Swindon, United Kingdom; 20 Pakistan Psychiatric Research Centre, Fountain House, Lahore, Pakistan; 21 Institute of Psychiatry, Psychology & Neuroscience, Kings College London, London, United Kingdom; PLOS: Public Library of Science, UNITED KINGDOM OF GREAT BRITAIN AND NORTHERN IRELAND

## Abstract

Trauma is a complex and often contentious psychopathological construct. The term trauma has become ubiquitous within mental health literature and practice. It is often used interchangeably to describe the etiology and the reaction to it. In this article we describe its historical and contemporary conceptualization through a review of the disorders that claim a direct relation to traumatic events whether or not they are recognized by official psychiatric classification systems. We critically evaluate the extent to which current understandings of traumatic stress disorders capture the diversity and complexity in trauma experiences and responses across global contexts. Post Traumatic Stress Disorder continues to be the most used clinically and most studied academically. Other diagnoses such as Ongoing Traumatic Stress Reaction and Continuous Traumatic Stress are becoming more prevalent in psychiatry, and simultaneously, Complex PTSD is challenging the way we perceive and address some personality disorders. A realignment of the definition among the various mental health professions, in addition to a comprehensive evaluation of the relevance of current classification for the nature and timeline of traumatic events, in particular in war times, would ensure better research, interventions, and, ultimately, outcomes for individuals and communities affected by traumatic events.

## Introduction

Trauma and its related concepts of stress and traumatic stress have been central to the conceptualization, understanding and treatment of human psychopathology. Since the inception of mental health care, and even before the existence of the distinct professional fields of psychiatry and psychology, most dysfunctional mental states or behaviors have been blamed on experiences and injuries, not too dissimilar to other conditions in the medical field. The etymology of trauma is complex and is believed to be of Indo-European roots meaning ‘to perforate’ [1] and/or of Greek origin meaning ‘wound’ [2]. Scientific advances and social transformations have led to evolutions in the definition of trauma and its manifestations at the individual, group, and community levels [2]. It was originally and continues to be used in reference to physical injuries, with the psychological dimension appearing much later in the twentieth century. In the early days, traumatic stress symptoms were assumed to be predominantly due to a flawed character, rather than a response to a traumatizing experience [3]. The DSM (Diagnostic and Statistical Manual of Mental Disorders) has played a central role in the evolution of psychiatry. Being the foundation of Western psychiatry and a key reference for health professionals and clinicians around the world, it serves as one of the most important developments in the diagnosis and management of mental health conditions.

The first traumatic stress disorder to be formally recognized was Post Traumatic Stress Disorder (PTSD) in 1980 [4]. DSM-1 and DSM-2 did not make any specific reference to trauma [5] although it did conceptualize of a relation between exposure to a situation and certain psychiatric pathology. Over the years other disorders were either hypothesized or observed. Some of these reached the threshold of acceptance by the psychiatric academic community and the World Health Organization (WHO) that jointly act as the validating authority of diseases through inclusion in the DSM and the International Classification of Diseases (ICD). The latest version of the ICD [6] featured a category dedicated to the stress disorders. In addition to the already-established PTSD and Adjustment Disorder, it integrated the novel diagnoses of complex Post traumatic Stress Disorder (cPTSD) and Prolonged Grief Disorder [7], while Acute Stress Disorder (ASD) was effectively downgraded to a ‘factor influencing health status’. The DSM-5, preceding the ICD-11 published in 2013 remained more conservative in its revision avoiding these novel diagnoses and retaining ASD [8]. This was maintained in the subsequent 2022 DSM-5-TR version.

In parallel, some influential circles in the field of psychology have taken a different route altogether in relating adult suffering to early life adversity under the broad heading of ‘trauma’. This culminated recently with the school of ‘trauma-informed therapy’, which aims to recognize the widespread impact of trauma and to create sensitive, safe and supportive environments for individuals seeking healthcare in order not to risk inflicting further psychological harm [9].. The conceptual frameworks of Ongoing Traumatic Stress Reaction and Continuous Traumatic Stress have also made an appearance in the literature without reaching a wide audience. The aim of this review is to critically evaluate the extent to which current understandings of traumatic stress disorders capture the diversity and complexity in trauma experiences and responses across global contexts. It is an attempt by a group of academics and clinicians to revisit the history and current position of the formal and less formal trauma disorders in circulation, with an emphasis on their inter-relation and their applicability to research and clinical practice. Their validity on a global scale is also discussed from a geopsychiatry perspective, especially in the context of political violence, armed conflicts and disasters.

### Post traumatic stress disorder

The idea that adverse life events can have an effect on our mental wellbeing goes way back in history. Ancient writers, such as Homer narrating Achilles in the *Illiad*, described combat stress reactions similar to what we know today as PTSD symptoms. During the First World War, the belief that neuropsychiatric changes in soldiers were the result of the impact of war exposure on their central nervous system led to the development of the ‘shell shock’ concept [10]. In the wake of the Vietnam War, due to the resemblance of post-combat symptoms between soldiers from this war and previous wars, researchers argued that PTSD symptoms are generalizable to all combat veterans [11]. Researchers subsequently reported PTSD symptoms in non-combat personnel who served in Vietnam. This was a first step before the expansion of the diagnosis to the civilian realm. Over time, a broader range of potentially traumatizing events started gaining recognition in the literature, in parallel to more flexibility in recognizing the diverse ways trauma can present [11].

The official diagnosis of PTSD was introduced in the 3^rd^ edition of the DSM in 1980 [4] in the anxiety disorders section. This introduction was due to extensive evidence, in light of the post-Vietnam syndrome, that stress disorders following traumatic events were found among non-combatant civilians and had a common phenomenology manifested through three categories of symptoms: reexperiencing, autonomic or cognitive symptoms, and numbing of responses [12]. DSM-5-TR states that exposure to ‘actual or threatened death, serious injury, or sexual violence’ is necessary for a PTSD diagnosis that manifests through three symptom clusters: re-experiencing, avoidance, and a perceived sense of current threat [13].

Studies conducted in various countries worldwide, such as Japan, Colombia, Iraq, Brazil, Mexico, South Africa, Australia, and Lebanon have provided evidence supporting the prevalence and clinical validity of PTSD across cultures [14]. The lifetime prevalence is reported to be 3.9% among the general population, and ranges from 5.6% [14] to 36% among trauma exposed individuals [15]. The consensus is that most individuals faced with a traumatic experience will process it naturally without the need for intervention, while a minority of individuals go on to develop PTSD.

Several demographic factors have been associated with increased lifetime risk of PTSD, including lower socio-economic status, in addition to being young, female and single [14]. A recent systematic review revealed a significantly higher prevalence of PTSD among asylum seekers, refugees, and people living in war zones, compared to individuals exposed to other types of trauma [15,16]. While the original diagnostic model for PTSD centered around responses to extreme life-threatening events, current clinical and research discourse acknowledges that PTSD symptoms may arise within broader political and socio-cultural contexts, such as racism, poverty, forced displacement and systemic violence. This challenges the universal applicability of the original Western biomedical model of PTSD and reflects a shift from viewing trauma as an isolated incident to viewing it as embedded in people’s lived experiences. This also calls for greater attention toward the cultural and contextual significance of suffering, as culture can even shape what is experienced as normal and what disrupts a person’s life [17]. In fact, since its inception, PTSD has been criticized for its cross-cultural validity with concerns about its applicability to diverse populations worldwide [18]. The criticism mainly suggests that some responses that are not included in European and American diagnostic frameworks may serve as hallmarks of trauma reactions in certain cultures, that culturally specific interpretations of suffering influence the way people experience and express distress, and that groups facing persistent adversity may not conform with current biomedical classifications like PTSD [18–20]. There has been recent criticism from scholars about the exportation of the PTSD discourse to the Arab region and its imposition on communities that significantly differ from the Western populations where it was initially developed and implemented [21,22]. For example, in the Middle East, trauma is often somatized and expressed through physical symptoms such as chronic pain, fatigue, or cardiovascular complaints, rather than through cognitive-emotional symptoms emphasized in Western classification [23]. Additionally, studies from Palestine and Lebanon show that individuals exposed to continuous conflict often frame suffering in collective, political or spiritual terms, viewing trauma as a shared social injustice rather than an individual psychological disorder [24,25]. These recognized differences may significantly influence the estimation of the pathologizing effect of war, conflict and displacement and undermine efforts to address it [26].

In terms of clinical intervention, Trauma-Focused Cognitive Behavioral Therapy (TF-CBT), a structured short-term intervention centered around the cognitive reprocessing of traumatic events and the development of coping skills to manage trauma reactions [27], is the most evidence-based treatment for PTSD in children and adults [28,29]. Other therapies such as Eye Movement, Desensitization, and Restructuring (EMDR), based on reprocessing traumatic memories through bilateral brain stimulation and side to side eye movements, and Narrative Exposure Therapy (NET), based on creating a chronological meaningful narrative of one’s life with integrated traumatic memories, have also been shown to be effective [29].

Although TF-CBT has demonstrated efficacy in treating trauma across various settings, its direct application in non-Western cultures raises critical concerns, such as in contexts of ongoing threat [30]. As this intervention is based on cognitive reframing and centers around fostering individual autonomy, it may not apply to communities where trauma is embedded in collective history and ongoing systemic violence where healing is often considered communal. Attempts have been made to create culturally adapted versions of TF-CBT with diverse populations such as in China, Japan, Jordan, DRC, Haiti and Northern Iraq, with most attempts adapting the intervention to suit the local culture through language translation and activity and analogy modification. A systematic review revealed weaknesses in the majority of studies, which could influence treatment efficacy and fidelity to the original protocol [31]. It also highlighted how therapies based on contemporary occidental cultural frameworks may not only be misunderstood but also frankly distrusted by individuals from other cultures. Western trauma programs have also been shown to be considered out of context by local practitioners in Middle Eastern contexts [22]. For instance, standardized protocols may overlook collective understandings of suffering and culturally specific idioms of distress, as well as traditional practices that may play a crucial role in recovery for certain populations. These therapies may therefore reinforce injustice by marginalizing non-Western conceptualizations of trauma, which calls for the development of new practices grounded in contextual understandings of trauma.

### Acute stress disorder

The diagnosis of ASD was first recognized as a disorder in 1994 with its inclusion in the DSM-IV. It is a diagnosis meant to have a clear temporal relation to an acute traumatic event and lasting 4 weeks after exposure. Its equivalent diagnosis in the ICD-10 was acute stress reaction (F43.0), which was later downgraded to a factor affecting health status (QE84) [7]. It receives less attention than other trauma-related disorders in the literature [32] in part due to the difficulty in researching short-term illnesses, especially if they resolve spontaneously, following a brief intervention or morph into other disorders such as PTSD, generalized anxiety, depression or even a psychotic disorder.

While initially believed to predict PTSD, for instance in victims of violent crimes [33] or witnesses of such crimes [34], later studies revealed at best a moderate predictive value for a chronic post-traumatic reaction. In other words, most individuals who present with ASD do not go on to develop PTSD [32]. Its prevalence in times of war is poorly understood for this same reason, in addition to the absence of access to psychiatric assessment and care during active conflict. Studies from recent war zones reveal a high level of incidence, including 93% in a sample of Ukrainian refugees [35]. Other studies reveal a more complex picture with a wide range of factors impacting incidence of stress reaction and stress disorder, including age [36] and personality features in addition to the degree of war exposure [37]. In combat situations, a distinction has been made between acute stress reaction and ASD with the former being found to be not necessarily predictive of the latter. A prevalence of 17% of acute stress reactions emerged from a study in the US military [38]. Interestingly, no gender difference was found. A recent study from Lebanon also reported a 38.4% prevalence of probable ASD among healthcare workers following the Beirut Explosion in 2020, with key predictors being of female gender, witnessing dead or mutilated bodies, and experiencing the death of a close one [39].

When it comes to determining effective interventions, the overlap between ASD and PTSD within a relatively brief timeline has meant relatively little interest in treating the former versus preventing the latter. TF-CBT was found to have a protective effect for further developing PTSD once an acute stress reaction was detected [40]. A systematic review for preventative pharmacological treatments within hours to days following exposure determined a role for beta-blockers and corticosteroids, although routine administration could not be recommended [41].

The time-bound symptom focused model of ASD has been criticized for risking the pathologization of transient stress reactions that, as previously discussed, are not necessarily predictive of future pathology and PTSD [42]. Furthermore, its requirements for specific symptom clusters such as dissociation has been considered overly restrictive as it may not recognize the heterogeneity of early posttraumatic stress responses and may not account for different cultural manifestations of responses to trauma [43]. It may thereby risk missing early signs of distress in populations whose experiences do not conform with its diagnostic criteria. This calls for a need to expand ASD’s contextual framework to better reflect different global factors that shape acute trauma responses.

### Continuous traumatic stress

The concept of continuous traumatic stress (CTS) was first introduced by mental health activists in response to state oppression and political violence in 1980s South Africa [44]. The group noted that victims in South Africa were living under chronic oppressive circumstances and that constant anticipation of future threat was a valid response [44]. It was thus hypothesized that the manifestation of their symptoms differs phenomenologically from those of PTSD, which centers on past memories intruding into the present after the threat had subsided. CTS conversely centers on present and future trauma exposure, rather than on a defined past exposure.

There is increasing recognition that the psychiatric impact of continuous exposure to traumatic stress is not captured in current psychiatric classification systems [45–47]. In both complex and classic PTSD, the traumatic experience is conceptualized to belong in the past relative to the time of diagnosis. Research has shown that reactions to continuous exposure to threat are broader and more intense compared to those associated with a single past traumatic event [48,49]. This threat is also not necessarily of a physically violent nature, as it can extend to situations such as a sense of chronic lack of social or economic safety, and living in a country with high rates of corruption [50].

While research in the domain of CTS remains preliminary, studies have shown that PTSD may not be accurate for representing the psychological profiles of individuals exposed to CTS [46,51]. There has been criticism in the literature around using the PTSD conceptual framework and PTSD measures to study the psychopathological manifestations of CTS, as had been the case historically [52]. The primary criticism centers on the fact that avoidance, hyperarousal, and intrusion may actually be adaptive and protective in the context of CTS, as opposed to maladaptive and ‘false alarm’ as conceptualized in PTSD, as threat and harm may be realistic and imminent in these contexts [45,53]. For instance, an individual living under war might need to be constantly alert and ready to run immediately in case of a siren onset, bombing warning, or hearing a warplane approaching.

When it comes to impaired functioning, which is diagnostically required for PTSD [13], research has shown that adaptive functioning can remain in contexts of CTS and may be the most common profile observed [51,53]. Studies have revealed that symptomatic resilience in the face of CTS may be more common than low, moderate, or high symptomatology [51]. This may be due to the individuals habituating to living under such circumstances or accepting living under occupation [54–56]. This may also be due to the fact that faith, along with social and community support, can help people in remaining resilient in the face of these CTS conditions [54,56]. Patriotism and a belief in resistance may also be factors facilitating their resilience [56,57].

In the literature examining the specific phenomenology of CTS, there is certain overlap with the behavioral, cognitive, and emotional manifestations of PTSD, such as constant concern for the future, depression, suicidal ideation, somatization, helplessness, and anxiety [58–61]. Additional symptoms include a sense of lack of protection, a low frustration threshold, and mental exhaustion [47]. There has been some evidence that psychopathology spikes during periods of escalations then decreases relatively fast when the environmental situation improves [58]. Eagle & Kaminer [45] also argued that the CTS phenomenon is likely to remit spontaneously if the person is able to escape the continuous traumatic situation.

Conflicts and situations of continuous trauma exposure are prevalent globally, and have been on the rise [62]. At the time of writing, several countries are experiencing situations of continuous or protracted armed conflict, such as Palestine, Lebanon, Syria, Yemen, Libya, Sudan, Ukraine, and Russia. This has sparked a renewed interest among mental health professionals and conflict trauma scholars in researching the concept of CTS over the last two decades [45,48,49], and more recently in light of the active wars in Ukraine and Palestine [63,64]. Situations of continuous trauma exposure can also be extended to living under state oppression, in a prison environment [65] or under threat of terror attacks [66]. This growing recognition that traditional trauma models are not representative of populations living under continuous traumatic situations challenges current dominant diagnostic frameworks by proposing a new framework that is more reflective of their realities.

### Ongoing traumatic stress reaction

Ongoing traumatic stress reaction (OTSR) for individuals under threat from armed conflict was first conceptualized by Diamond et al. in 2010 [67]. They described a presentation similar to CTS characterized by a gradual increase in anxiety, an anticipation of future imminent attacks, hyperarousal (e.g., waiting for a missile attack siren warning), realistic fears and reality-based patterns of avoidance. These symptoms, like in CTS, undergo a substantial reduction or complete resolution soon after the risk of danger is considered to have subsided. This could be during a ceasefire, truce or peace agreement. The authors believed that OTSR could lead to impairment in functioning and quality of life, through avoidance and isolation leading to a decrease in social support and depression, and prolonged hyperarousal leading to chronic insomnia. However, they also considered that these reactions are not inherently pathological and could be adaptive in what are abnormal circumstances [56,67]. They thus suggested that OTSR is intended to address a clinically meaningful, but not necessarily pathological, presentation.

The authors noted that even though OTSR may not qualify as a psychiatric disorder, intervention may still be necessary in the form of psychoeducation, validation of the reality based stress reactions, along with interventions for people experiencing OTSR which can be useful in terms of focusing on managing day to day anxiety and coping skills, and planning exposures to fear evoking situations, such as re-engaging in daily activities that have been avoided as a result of ongoing threat while still prioritizing safety. Two systematic reviews also revealed that culturally adapted trauma-focused psychological interventions, mainly TF-CBT, appear to be promising and may be feasible and beneficial in contexts of ongoing threat, with cautions due to the paucity, heterogeneity, and poor quality of existing studies [29,68]. While these reviews do not explicitly refer to OTSR, the majority of the studies included populations exposed to ongoing traumatic threat from armed conflict. This suggests that these interventions may hold relevance for populations experiencing OTSR, highlighting the need for more targeted research in this area.

Research on OTSR remains limited despite the rising prevalence of ongoing traumatic stress situations, similarly to CTS [69]. Few attempts have been made at systematically distinguishing between OTSR and PTSD beyond anecdotal reports from clinical observations, and OTSR remains a developing construct without formal diagnostic recognition [67,69]. There is also an obvious overlap between the concepts of OSTR and CTS that has not yet been formally addressed.

### Complex post traumatic stress disorder

cPTSD has emerged as a distinct diagnosis that describes the psychological consequences of prolonged, repeated, and interpersonal trauma, including childhood abuse, intimate partner violence, captivity, torture, or exploitation [70,71]. Although clinicians have long recognized the inadequacy of the PTSD framework to fully capture the breadth of trauma-related psychopathology in such cases, the formalization of cPTSD was finally achieved with its inclusion in the 11th edition of the International Classification of Diseases (ICD-11) published by the WHO in 2018 [6,7].

According to ICD-11, cPTSD consists of a core symptom cluster — re-experiencing, avoidance, and a persistent sense of current threat — combined with a syndrome of Disturbances in Self-Organization (DSO). This includes:

1. Affective dysregulation, such as emotional numbing or heightened emotional reactivity
2. Negative self-concept, including persistent beliefs of worthlessness, shame, or guilt
3. Interpersonal difficulties, including avoidance of relationships or inability to maintain closeness [70,72]

It is primarily the presence of DSO in an individual that clinically distinguishes cPTSD from PTSD in the ICD-11 and reflects empirical research on symptom profiles among survivors of complex trauma [73].

While the ICD-11 explicitly includes cPTSD as a separate diagnosis under “Disorders Specifically Associated with Stress”, the DSM-5, published by the American Psychiatric Association (APA) in 2013, does not recognize cPTSD as a distinct category. Instead, it broadened the diagnostic criteria for PTSD to include symptoms related to mood, cognition, and dissociation [8,74]. However, proponents of cPTSD have argued that these expansions fail to provide sufficient clinical utility to differentiate between PTSD and more pervasive traumatic responses [75].

This divergence between classification systems has practical implications for diagnosis, treatment planning, and research, particularly in settings where the ICD-11 and DSM-5 are used concurrently [7]. It also hampers the development of internationally comparable epidemiological data as well as the advancement of tailored therapeutic approaches [2].

Several cross-national studies have demonstrated that cPTSD is a prevalent and valid clinical construct, especially among populations exposed to chronic or repeated interpersonal traumas. A latent class analysis of trauma-exposed individuals from different demographic backgrounds including Caucasian, Asian, Hispanic, and African-American, confirmed that cPTSD represents a distinct symptom profile rather than a more severe version of PTSD [70]. In refugee populations, studies conducted in host countries, including Lebanon and Switzerland, have reported cPTSD prevalence rates of 10–30%, with even higher rates when cumulative or early life trauma is considered, highlighting the compounding effect of repeated trauma exposure [73,76]. These studies included refugees from diverse backgrounds, including Syrian, Turkish, Iranian, Sri Lankan, Afghani, Bosnian, and Iraqi populations displaced as a result of war, torture, persecution, deprivation, community violence, natural disasters, or interpersonal violence.

The hallmark of treatment for cPTSD is a phase-based psychotherapeutic model, beginning with emotional stabilization and skills-building, followed by trauma-focused therapy, and ending with reintegration into social roles [77,78]. Interventions such as STAIR-NT (Skills Training in Affective and Interpersonal Regulation – Narrative Therapy), designed to help individuals build emotional regulation and interpersonal skills while also working on reintegrating traumatic memories into the individual’s life narrative, have demonstrated efficacy in reducing both PTSD and DSO symptoms and improving functional outcomes [79]. The role of pharmacotherapy remains unclear [80]. Recent efforts have focused on understanding the neurobiological correlates and dimensional structure of cPTSD, with the aim of personalizing care and identifying specific biomarkers of treatment response [81,82].

Although representing an important shift in acknowledging and examining repeated and prolonged trauma exposure, cPTSD research is still mostly limited to high-income countries and has not explored in depth the relevance of cPTSD in trauma survivors globally, particularly in the context of armed conflict, political violence, displacement, and extreme poverty. Further research is needed to examine the implementation of this diagnosis in different cultures and contexts and its clinical validity and utility in situations where variations of symptom profiles can be expected.

## Discussion

This overview of trauma related diagnoses and conditions reveals an evolving panorama influenced by clinical reality and academic research with PTSD as the core building block. This is despite the longstanding concerns of the validity of this model of trauma response outside the modern western ethnocultural experience. In fact, it appears that these recognized and postulated trauma diagnoses have also inherited the flaws associated with PTSD in terms of generalizability on a global scale. The justifiable attempts at broadening the scope of the traumatic experience through diagnostic constructs such as CTS and OTSR and the traumatic response through others such as cPTSD still suffer from a lack of large-scale validation and doubts over clinical utility. This is most felt in contexts where trauma is inflicted on a mass level in unstable geopolitical ecosystems where armed conflict, political violence, gross economic and ecological stress prevail. While this review draws primarily on research conducted in Western and high income contexts, this reflects a broader gap in trauma research globally, as low and middle income countries (LMICs) remain underrepresented in theoretical frameworks and empirical studies despite holding a substantial burden of trauma globally. This limitation highlights our argument for greater global inclusivity in trauma discourse, which is urgently needed for the development of culturally and contextually grounded models of trauma. This inclusivity can help in broadening the understanding of trauma experiences in different contexts, and can help inform interventions better attuned to the contexts in which this trauma occurs, as most current models are developed in high income and western countries with some efforts to then validate these constructs in non western settings.

Although trauma is part of the universal human experience, ethnocultural factors can shape what is considered trauma, the way it is interpreted and the way it manifests. In recent years, the mental health literature has noted the semantic inflation of the concept of trauma in clinical and popular discourse, calling it a “concept creep” [2,83,84]. This may be linked to more relaxed DSM criteria for what is considered a traumatic event over the years, as earlier editions required events that fall outside of usual human experiences and would evoke distress in most people, while later editions broadened the criteria to include events experienced indirectly or developmentally inappropriate events that are not necessarily life threatening [84]. While the growing recognition of trauma reflects a cultural shift in terms of increased awareness and validation of suffering that would have previously gone unrecognized, it has been argued that this broadening can blur the boundaries between discomfort and traumatic exposure thereby inciting over sensitivity to normal discomfort or less than-than-ideal life events and trivializing profound, life-threatening, or prolonged traumatic experiences [83]. It can also pathologize normal emotional responses to threatening life events, encouraging individuals to interpret typical life challenges as symptoms of a psychiatric illness. The term trauma thus loses its clinical precision and utility, risking the divergence of resources from those with most pressing needs and the inflation of the rates of traumatic stress disorders. Blehm [85] argues that ‘trauma is differentiated from other emotional experiences in that it includes one’s stress response triggered by an intense negative emotion like terror or horror and trauma also includes a disruption in the way one normally connects one’s identity with one’s experience’. Paradoxically, according to this definition that emphasizes the response rather than the trigger, trauma can result from various life changes, ranging from extreme situations to significant life events [86]. Extreme situations involve exceptional, highly stressful experiences, resulting from exposure to war, terrorism, abuse or natural disasters, and are generally more distressing and more overwhelming in terms of coping capacity than typical life events. Yet, even this threshold for a ‘traumatic experience’ is subject to debate. Through the process of habituation, what is traumatic in one context becomes a typical life in another. The importance of a variable threshold for trauma induction contributes to the understanding of resilience in populations subjected to extreme conditions over a protracted period. Its interplay with individual psychological vulnerability and socio-cultural dogma is still poorly understood. Ultimately, while the broader recognition of trauma has brought important benefits, its conceptual clarity must be protected in order to ensure that the term retains its meaning and clinical utility.

A review of DSM and ICD classifications of traumatic stress disorders reveals significant divergences with political, clinical, and research implications. While the DSM-5 presents a highly specific and symptom focused definition of PTSD which highlights fear based responses to discrete traumatic experiences, the ICD-11 presents a more streamlined approach with fewer symptoms and introduces cPTSD which accounts for disturbances in self-organization, offering a broader lens for developmental and chronic trauma. When it comes to research, the divergences between these two classification systems can complicate cross national studies, systematic reviews, and meta-analyses, and can limit the development of homogenous international diagnostic and therapeutic protocols. Clinically, this divergence may lead to inconsistencies in diagnosis and access to treatment across healthcare systems depending on which classification system is used. Politically, these divergences reflect different institutional priorities. The ICD, shaped primarily by a WHO global health framework, tends to prioritize clinical utility through more descriptive diagnoses intended for use by all healthcare professionals, while the DSM, shaped primarily by US psychiatric institutions and receiving more funding, tends to prioritize research utility and categorization for primary psychiatric use [87]. These differing priorities can in turn impact which disorders and syndrome presentations receive funding and insurance recognition, and can contribute to unequal access to care and to the marginalization of non-western conceptualizations of trauma. These divergences not only highlight diagnostic and therapeutic challenges, but also reflect deep rooted differences in how trauma is conceptualized and addressed across global contexts.

We reviewed PTSD, ASD, and cPTSD, all trauma-related disorders arising either from a single or from repeated past trauma experience. In terms of symptomatology, the distinction between PTSD and cPTSD is the clearest with the adoption of a characteristic cluster specific to cPTSD. Table 1 presents a side-by-side comparison of both disorders as defined in ICD-11 [6,7,70].

**Table 1:** Diagnostic Criteria of PTSD and Complex PTSD According to ICD-11.

Criterion	PTSD	Complex PTSD (cPTSD)
**Trauma Exposure**	Exposure to extremely threatening or horrific event(s)	Prolonged or repetitive trauma (often interpersonal), such as childhood abuse, captivity, torture
**Core PTSD Symptoms**	Re-experiencing, avoidance, and sense of current threat	Same core PTSD symptoms
**Additional Symptom Cluster**	None	Disturbances in Self-Organization (DSO): affective dysregulation, negative self-concept, and interpersonal difficulties
**Functional Impairment**	Present due to core PTSD symptoms	Present due to both PTSD and DSO symptoms
**Diagnostic Classification**	ICD-11 and DSM-5 (with some variation)	ICD-11 only
**Treatment Implications**	Trauma-focused therapy (e.g., CBT, EMDR, NET)	Phase-based treatment: stabilization, trauma processing, and reintegration; may include STAIR–NT

The cross-cultural generalizability of this DSO symptom cluster is not yet established. As the current cPTSD literature remains mostly limited to high income countries, further research is needed to examine how complex trauma influences personality development and the experession of DSO symptoms across diverse global contexts. In addition, although the International Trauma Questionnaire (ITQ) has shown good psychometric performance across diverse settings, including Europe, South America, Africa, and the Middle East [72,88], further research is needed to clarify how the sociocultural context and language shape the phenomenology and interpretation of cPTSD symptoms [80]. For instance, validating a scale in one African context, such as validating the ITQ in Nigeria or Kenya [89], cannot be assumed to be applicable to the continent in all its diversity or even to just the Sub-Saharan component. The same issue arises when considering Latin America, the Arab world, the Caribbean, South East Asian or even the Indian subcontinent. A common language, religion or national identity does not necessarily equate with shared psychosocial characteristics. Without the involvement and leadership of local scholars and advocates, simplistic ethnocentric assumptions fall in the trap of overlooking essential historical, linguistic, and cultural diversity that can only be truly appreciated from within.

cPTSD has been studied in group populations, such as in racial minorities and in refugees. Racial trauma (i.e., microaggressions, verbal and physical attacks, death threats, being beaten, and witnessing racist homicides) has been paralleled to complex trauma in Black, Indigenous, and People of Color (BIPOC) in North America, as both traumas may share similar interpersonal, biological, cognitive, and socioeconomic consequences, with major long term health effects [90]. Carter et al. [91] argued that racial trauma should be considered inherently complex due to its pervasive and cumulative nature and its harmful consequences throughout BIPOC’s lives. In refugee populations, cPTSD prevalence rates have been found to be higher than those found in community samples [76,92]. cPTSD prevalence rates have also been shown to be higher than PTSD prevalence rates in treatment seeking refugees [93,94].

From another angle, questions have been raised on the overlap in clinical presentation between Borderline Personality Disorder (BPD) and cPTSD [95]. Clearly framing symptoms in relation to a traumatic etiology could lead to the adoption of cPTSD as a less stigmatizing diagnosis than BPD [92,95]. Researchers note some distinguishing features between the two, such as the presence of a consistently negative view of the self in cPTSD versus an alternating sense of self in BPD [92,96]. To aid clinical differentiation, a side-by-side comparison of core features of BPD and cPTSD is presented in Table 2 [70,92,96,97].

**Table 2:** Clinical Comparison Between BPD and cPTSD.

Criterion	BPD (DSM-5)	cPTSD (ICD-11)
**Etiology**	Multifactorial; often includes early relational trauma	Prolonged and interpersonal trauma (e.g., abuse, captivity, torture)
**Trauma requirement**	Trauma may be present but not required	Trauma is required for diagnosis
**Sense of self**	Unstable or fragmented identity	Persistently negative self-concept (e.g., shame, worthlessness)
**Affect regulation**	Intense and reactive emotional states	Chronic emotional dysregulation (e.g., hyperactivation or numbing)
**Interpersonal relationships**	Intense, unstable relationships; fear of abandonment	Difficulty establishing or maintaining relationships
**Impulsivity**	High (e.g., self-harm, substance use, risky sex, spending)	Not a core feature
**Dissociation**	Present, often during stress	May be present, but less central
**Self-harm/suicidality**	Frequent and part of diagnostic criteria	Can occur, but not core to the diagnosis
**Course**	Fluctuating, reactive to relational stressors	More stable and persistent if untreated
**Main treatments**	DBT, MBT, psychodynamic psychotherapy	Phase-based treatment (e.g., STAIR–NT)
**Recognized by**	DSM-5 and ICD-11	ICD-11 only (not in DSM-5)

Furthermore, there have been calls towards placing these disorders on a continuum in accordance with the dimensional model of psychopathology, with BPD being on the more severe end of the spectrum [96,97]. Variations in BPD symptom presentations exist across cultures, such as differences in self harm methods used, differences in impulsive behavior that may involve sex or drugs, and differences in rates of behaviors such as higher rates of interpersonal problems in Western societies [98,99]. Clinicians from some cultures, such as Asian cultures, have also shown skepticism with regards to the diagnosis of BPD [100]. These factors may lead to a misdiagnosis of BPD when a patient’s cultural context is not taken into account. This further indicates a need for a more dimensional approach when assessing BPD to improve identification and reduce cultural bias.

The contemporary psychiatric model of trauma is not adequate for capturing the full spectrum of traumatic experiences and symptomatology present in conflict-affected regions. This calls for caution in applying these concepts beyond industrialized Western societies, and for a need for a decolonization of the current conceptualization of trauma. This highlights a need for mental health professionals working in conflict settings to be well trained and equipped to deal with the distinctive needs of populations affected by conflict [101]. Clinical and research findings suggest that the phenomenology of PTSD and other aspects of complex, continuous or ongoing trauma exposure are shaped by cultural and contextual factors. For example, aspects of control and autonomy may be more prominent symptom presentations in individualistic cultures [102]. Further, there is evidence of important cultural differences in the management of emotional dysregulation associated with PTSD [103]. In collectivist cultures such as in Arab countries in the Middle East, community, social and familial support may play a protective role when coping with both past or ongoing war-related trauma [56]. Cultural competence amongst mental health professionals working in conflict settings is therefore key and should aim to avoid pathologizing adaptive responses to ongoing trauma, and to develop context appropriate effective treatment strategies. Current APA Clinical Practice Guidelines for individuals experiencing ongoing trauma highlight that safety planning and resilience building may be more appropriate to their needs than interventions designed for PTSD, and call for a need for the expansion of services offered in these contexts [104].

Another revelation from the review is the striking similarities between two emerging concepts: CTS and OTSR. These ‘syndromes’ have been proposed by distinct groups of scholars and activists to distinguish the effects of ongoing exposure to threat from the ‘post-trauma’ nomenclature. CTS has been proposed to arise in contexts where individuals face repeated or ongoing exposure to threat, such as in violent communities and war zones, and acknowledges that as the threat has not ended and there is no “post trauma”, individuals’ anxiety and hypervigilance may be adaptive responses to real threat rather that pathological symptoms present after the threat has ended. OTSR, proposed after CTS, builds on this by describing emotional and physiological responses triggered by the anticipation of future threat. Both CTS and OTSR conceptualize distress in ongoing trauma contexts not as signs of psychopathology, but as normal and adaptive responses to abnormal circumstances, unlike in other disorders such as PTSD, where symptoms are often interpreted as pathological reactions to past events. These two concepts are both largely grounded in limited theoretical and clinical observation, with limited robust empirical evidence, which may be largely due to the nature of the contexts in which these syndromes present and the difficulty in conducting mental health research in active conflict settings, with priority being given to survival and physical safety. Despite their growing relevance in populations experiencing continuous exposure to threat, large-scale studies aimed at validating or differentiating them remain limited. As their boundaries overlap, they have also been used interchangeably in research [69,105] and in the context of the COVID-19 epidemic and global lockdown, on the basis that its experience shares features with the traumatic effect of living through ongoing armed conflict [69,106,107]. While both CTS and OTSR challenge the limitations of a conventional linear ‘after-trauma’ diagnosis, CTS centers more heavily on the continued presence of external threat and the need for safety-focused interventions, whereas OTSR emphasizes internal emotional dysregulation in the face of normalized yet unresolved trauma. A comparison between these two concepts can be found in Table 3.

**Table 3:** Comparison of CTS and OTSR.

Feature	Continuous Traumatic Stress (CTS)	Ongoing Traumatic Stress Reaction (OTSR)
**Origin of Concept**	South Africa, post-apartheid	Recent Middle East conflict settings
**Core Focus**	Perception of persistent threat in high-risk environments	Emotional/cognitive reactions to an ongoing traumatic context
**Threat Status**	Threat is real, active, ongoing, or imminent	Ongoing but often ambient or cumulative stressors
**Temporal Focus**	Present and anticipated future trauma	Prolonged emotional and physiological activation
**Symptomatology**	Hypervigilance, avoidance, irritability, sleep disturbance	Anxiety, cognitive overload, helplessness, avoidance, isolation
**Contextual Assumptions**	No clear post-trauma phase	Trauma is normalized, part of everyday life
**Clinical Implication**	Calls for safety-focused, community-level interventions	Emphasizes emotional regulation and contextual grounding
**Sociopolitical Lens**	Strongly linked to structural violence and social justice	Emphasizes adaptation under persistent adversity

As the understanding of trauma continues to evolve to reflect the lived realities of populations in conflict-affected and chronically unstable environments, further research in diverse populations is urgently needed to examine whether sufficient evidence exists to differentiate the emergent constructs of CTS and OTSR in terms of scope, phenomenology, diagnosis and treatment pathways, in order to establish diagnostic clarity and inform targeted interventions. Both emerging frameworks highlight the importance of moving towards a structural and contextually grounded understanding of trauma in conflict-affected settings, rather than relying on static, event-based models. In settings where trauma is ongoing, rather than limited to events in the past, OTSR appears to offer a more clinically grounded approach than CTS which appears to be more powerful from a socio-political advocacy lens, offering a compelling human rights based approach that frames trauma as a consequence of structural violence.

Clinicians generally adopt the position that establishing safety and achieving a sense of psychological stabilization are essential before initiating trauma-focused therapeutic interventions, as psychological treatment aimed at processing trauma may pose the risk of causing harm for individuals undergoing ongoing threat [68,108]. As we seem to have entered a turbulent geo-political and economic era, where safety and stabilization cannot be assumed in certain contexts, there is urgent need for interventions developed or adapted for individuals experiencing ongoing threat, as current trauma focused interventions based on a post-traumatic framework are not fit for purpose. Interventions in these contexts need to be focused on helping clients assess real risk and develop strategies for relative physical and emotional safety rather than be focused on resolution and processing the trauma as in PTSD. Clients may therefore need support in distinguishing when their responses are protective rather than detrimental through encouraging situational awareness without pathologizing necessary vigilance. Interventions for CTS and OTSR should aim at strengthening emotional regulation strategies, coping resources, and flexible problem solving in unstable and unpredictable environments. As these syndromes may be rooted in systemic contextual conditions, standalone individual therapy may not be sufficient and may need to be supplemented by social support, advocacy, and community based interventions. In summary, interventions of CTS and OTSR need to be more tailored to the context in which they arise and to the needs of the populations that experience them. They thus need to be less focused on trauma-recovery and more focused on sustainability and adaptation in the face of ongoing trauma. Recovery may then be conceptualized to be an attempt to reconstruct a disrupted social context through empowerment, access to resources, social support, and cultural stability rather than an individual personal process [22]. The validity and utility for these interventions can only be established on the ground where they are most needed: Primarily in conflict zones, using a local workforce and for underserved populations.

## Conclusion

This review provides an additional perspective on the inadequacies of the trauma-related disorders despite the evolution of the field in general and the classification systems more specifically. The longstanding criticism of the global applicability of ASD and PTSD has not only been insufficiently addressed, but can now be extended to new diagnoses such as cPTSD and emerging concepts such as OTSR and CTS. Beyond the diagnostic overlaps and discrepancies that were highlighted in our paper, cross-cultural applicability continues to be an afterthought in the way all these disorders are conceptualized, researched and used in clinical practice. They particularly fail to adequately and inclusively capture the experiences of populations living under conditions of protracted oppression and violence. It is in conflict zones and in unstable geopolitical circumstances that most mass traumatic experiences take place. It is inconceivable that the discipline of trauma does not recognize this fact and use it to better understand the impact on the human psyche. This shortcoming reflects a deeper fundamental gap in global mental health that geopsychiatry seeks to bridge, by embedding the structural and geopolitical determinants of psychological distress into practice and calling for a fundamental shift towards universal context-sensitive diagnostic frameworks.
